# PKD1 is a potential biomarker and therapeutic target in triple-negative breast cancer

**DOI:** 10.18632/oncotarget.25292

**Published:** 2018-05-01

**Authors:** Caroline Spasojevic, Elisabetta Marangoni, Sophie Vacher, Franck Assayag, Didier Meseure, Sophie Château-Joubert, Martine Humbert, Manale Karam, Jean Marc Ricort, Christian Auclair, Marie Regairaz, Ivan Bièche

**Affiliations:** ^1^ Pharmacogenomics Unit, Department of Genetics, Institut Curie, Paris, France; ^2^ LBPA, CNRS UMR8113, ENS Paris-Saclay, Paris-Saclay University, Cachan, France; ^3^ Translational Research Department, Institut Curie, PSL Research University, Paris, France; ^4^ Department of Pathology, Institut Curie, Paris, France; ^5^ BioPôle Alfort, Ecole Nationale Vétérinaire d’Alfort, Maisons Alfort, France; ^6^ AB Science SA, Paris, France; ^7^ Cancer Research Center, Qatar Biomedical Research Institute, Hamad Bin Khalifa University, Qatar Foundation, Doha, Qatar; ^8^ Biology Department, ENS Paris-Saclay, Paris-Saclay University, Cachan, France

**Keywords:** triple-negative breast cancer, protein kinase D1, PKD, PKC

## Abstract

Protein Kinase D1 (PKD1) is a serine/threonine kinase encoded by the *PRKD1* gene. PKD1 has been previously shown to be a prognostic factor in ERα+ tamoxifen-resistant breast tumors and PKD1 overexpression confers estrogen independence to ERα+ MCF7 cells. In the present study, our goal was to determine whether PKD1 is a prognostic factor and/or a relevant therapeutic target in breast cancer. We analyzed *PRKD1* mRNA levels in 527 primary breast tumors. We found that high *PRKD1* mRNA levels were significantly and independently associated with a low metastasis-free survival in the whole breast cancer population and in the triple-negative breast cancer (TNBC) subtype specifically. High *PRKD1* mRNA levels were also associated with a low overall survival in TNBC. We identified novel PKD1 inhibitors and assessed their antitumor activity *in vitro* in TNBC cell lines and *in vivo* in a TNBC patient-derived xenograft (PDX) model. Pharmacological inhibition and siRNA-mediated depletion of PKD1 reduced colony formation in MDA-MB-436 TNBC cells. PKD1 inhibition also reduced tumor growth *in vivo* in a TNBC PDX model. Together, these results establish PKD1 as a poor prognostic factor and a potential therapeutic target in TNBC.

## INTRODUCTION

Breast cancer is currently the first cause of death from cancer in women, and the second most common cancer overall, with 1.7 million new cases and 521,900 deaths each year according to the most recent worldwide study [1]. Breast cancer prognosis is variable, depending mostly on tumor stage at diagnosis and on the molecular features of the tumor. Breast tumors can be divided into different molecular subtypes: i) the Luminal A and B subtypes, expressing high levels of estrogen and/or progesterone receptors, ii) the HER2+ subtype, overexpressing the human epidermal growth factor receptor 2 (HER2) protein and iii) the triple-negative breast cancers (TNBC), expressing none of the hormone receptors and showing no HER2 amplification and/or overexpression [2, 3]. TNBC and HER2+ cancers are the most aggressive tumors with the highest metastatic potential. The poor prognosis of TNBC also results from the lack of treatment options for these patients, who cannot benefit from either hormone or HER2-targeted therapies [4]. Although hormone-sensitive tumors can be treated with endocrine drugs, resistance is observed in about 40% of advanced stage cases [5]. Thus, it remains very important to identify new targets and associated biomarkers for breast cancer therapy.

We have previously shown that the Protein Kinase D1 (PKD1) can promote both proliferation and estrogen independence in breast cancer cells [6, 7]. PKD1 is a serine/threonine kinase encoded by the *PRKD1* gene [8]. PKD1 belongs to the PKD family (together with PKD2 and PKD3) within the CAMK (calcium/calmodulin-dependent kinase) superfamily. It is an atypical protein kinase C (PKC) activated by growth factors, mitogenic neuropeptides, as well as oxidative stress [9]. PKD1 regulates a variety of biological processes such as cell proliferation, survival, motility, organization of the Golgi apparatus and membrane trafficking [10, 11]. Hotspot activating mutations of *PRKD1* have recently been identified in polymorphous low-grade adenocarcinomas of salivary glands and likely constitute oncogenic drivers in these tumors [12]. In breast cancer, a study from Kim and coll. showed that PKD1 can induce chemoresistance in cells [13]. In addition, we have previously demonstrated that PKD1 can confer resistance to antiestrogen therapy in ERα+ breast cancer cells [6]. Thus, PKD1 is likely to be a relevant therapeutic target in breast cancer.

The objective of the present study was to determine whether PKD1 can be a prognostic factor and/or a therapeutic target in breast cancer. Because PKD3 has also been identified as a potential molecular target in breast cancer [14], we extended our study to the two other members of the PKD family. Thus, we first analyzed PKD1, PKD2 and PKD3 expressions in a large series of primary breast tumors. After identifying PKD1 as an independent prognostic factor in TNBC, we assessed the antitumor activity of PKD1 pharmacological inhibition in TNBC cell lines and patient-derived xenografts (PDXs).

## RESULTS

### *PRKD1* expression is a prognostic factor in total and TN breast cancer

**Figure 1:**
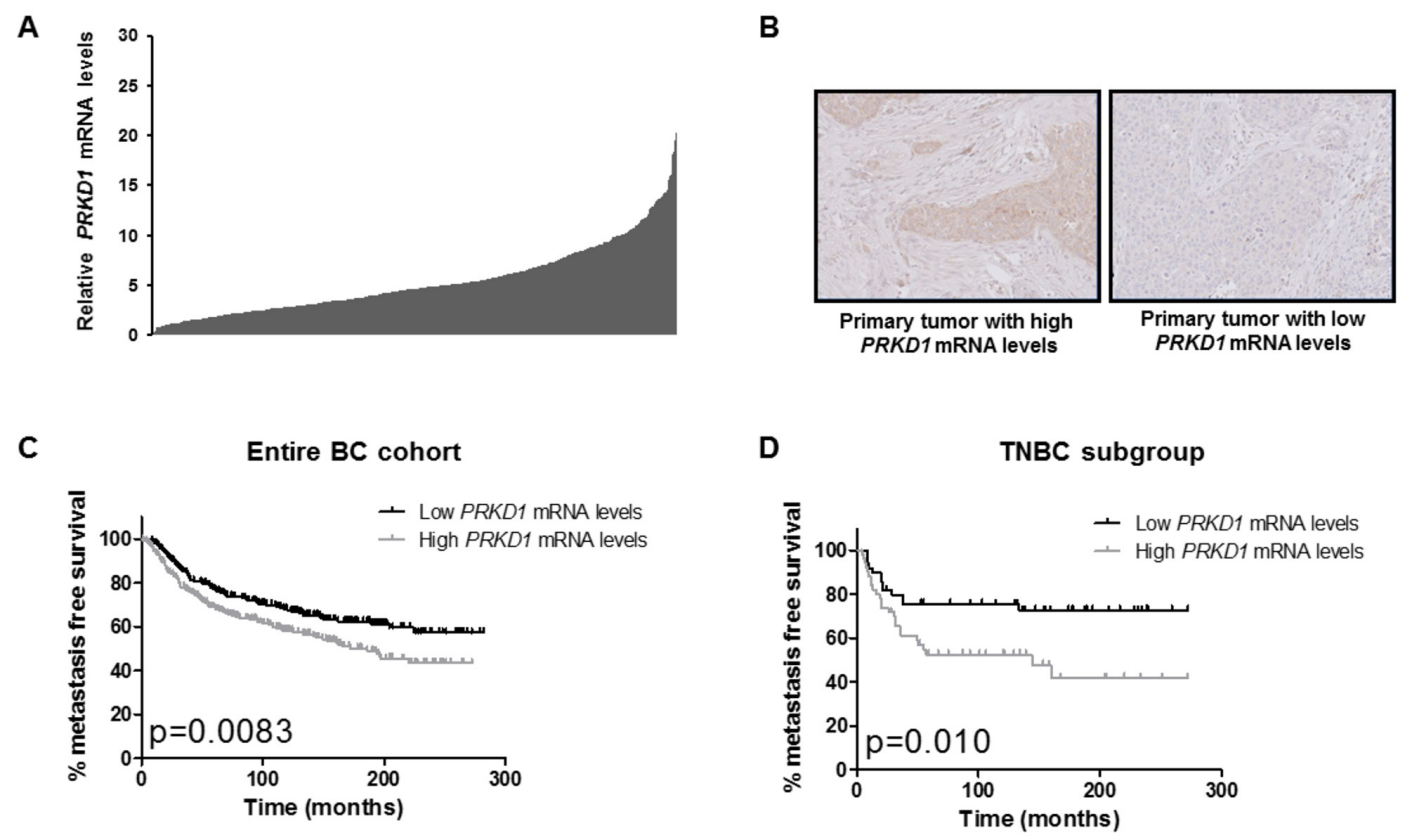
*PRKD1* expression is a poor prognostic factor in the entire breast cancer cohort and in the TNBC subgroup **(A)** *PRKD1* mRNA levels in 527 primary breast tumors. *PRKD1* mRNA expression was analyzed by RT-qPCR and normalized to that of the TBP control gene. Normal breast tissues were used as a reference (expression level =10). **(B)** Immunohistochemical analysis of PKD1 protein expression in primary breast tumors showing high or low *PRKD1* mRNA levels (relative *PRKD1* mRNA levels of 10.2 and 1.0, respectively). Original magnification x200. **(C)** Kaplan-Meier analysis of metastasis-free survival according to *PRKD1* mRNA expression in the entire breast cancer cohort (n=527). **(D)** Kaplan-Meier analysis of metastasis-free survival according to *PRKD1* mRNA expression in TNBC (n=102).

To determine whether the expression of PKD family members is associated with prognosis in breast cancer, we first analyzed *PRKD1*, *PRKD2* and *PRKD3* mRNA levels by quantitative RT-PCR in a large series of 527 primary breast tumors with known clinical/pathological status and long-term outcome (Figure 1A). The cohort was composed of 102 HR-/ERBB2- (TNBC), 72 HR-/ERBB2^+^, 295 HR+/ERBB2- and 58 HR+/ERBB2+ cases. Clinical, pathological and biological characteristics of the entire cohort are described in Table 1. In this cohort, a high histological grade, high pathological size, lymph node status >3 and negative progesterone receptor status were significantly associated with a lower metastasis-free survival (MFS) (Table 1).

**Table 1:** Clinical, pathological and biological characteristics of the 527 primary BC tumors

	Entire cohort (%)	Metastatic cases (%)^*g*^	*p-*value^*a*^
*Total*	527 (100.0)	210 (39.8)	
*Age*			
≤50	125 (23.8)	52 (41.6)	0.52 (NS)
>50	402 (76.2)	158 (39.3)	
*SBR histological grade*^b.c^			
I	60 (11.7)	12 (20.0)	**0.0013**
II	241 (47.1)	100 (41.5)	
III	211 (41.2)	94 (44.5)	
*Lymph node status*^d^			
0	159 (30.5)	48 (30.2)	**0.0000001**
1-3	250 (47.9)	88 (35.2)	
>3	113 (21.6)	72 (63.7)	
*Pathological size*^e^			
≤25mm	248 (48.0)	77 (31.0)	**0.0000055**
> 25mm	269 (52.0)	132 (49.0)	
*ER status*			
Negative	181 (34.3)	75 (41.4)	0.10
Positive	346 (65.7)	134 (38.7)	
*PR status*			
Negative	255 (48.4)	110 (43.1)	**0.025**
Positive	272 (51.6)	100 (36.7)	
*ERBB2 status*			
Negative	473 (89.8)	190 (40.2)	0.55
Positive	54 (10.2)	20 (37.0)	
*Molecular subtypes*			
HR-/ERBB2-	102 (19.3)	38 (37.3)	0.054
HR-/ERBB2+	72 (13.7)	36 (50.0)	
HR+/ERBB2-	295 (56.0)	115 (39.0)	
HR+/ERBB2+	58 (11.0)	21 (36.2)	
*Histological subtypes*^f^			
Apocrine	2 (0.45)	1 (50.0)	0.96 (NS)
Colloid	4 (0.90)	2 (50.0)	
Ductal	398 (89.6)	156 (39.2)	
Lobular	28 (6.3)	11 (39.3)	
Medullary	4 (0.90)	1 (25.0)	
Metaplastic	1 (0.23)	1 (100.0)	
Mixed	5 (1.13)	2 (40.0)	
Papillary	1 (0.23)	0	
Tubular	1 (0.23)	0	

^a^: Log-rank Test (MFS).

^b^: Scarff Bloom Richardson classification.

^c^: information available for 512 patients.

^d^: Information available for 522 patients.

^e^: Information available for 517 patients.

^f^: Information available for 444 patients.

^g^: Percentages of metastatic cases were calculated from the corresponding line in the entire cohort column.

*PRKD1* mRNA expression was detected in 99.8% of cases while *PRKD2* and *PRKD3* mRNA expressions were detected in all cases. Importantly, we were able to detect PKD1 protein expression by immunohistochemistry in five tumors expressing high *PRKD1* mRNA levels. Conversely, no PKD1 protein expression was observed in five samples showing low *PRKD1* mRNA levels (Figure 1B). In primary breast tumors, PKD1 protein expression was detected both in tumor cells and in cells from the tumor microenvironment, including fibroblasts, mononuclear immune cells and endocytes (Supplementary Figure 1A). PKD1 immunoreactivity was essentially cytoplasmic but PKD1 was also localized both in the cytoplasm and nucleus in some samples (Supplementary Figure 1B). It is noteworthy that PKD1 expression was also detected in normal breast tissues (Supplementary Figure 1D).

To assess the prognostic value of *PRKD1*, *PRKD2* and *PRKD3* expressions in our cohort, median expression levels were used as cutoff values to stratify patient samples intro groups of low expression and high expression. Outcome and clinical/biological parameters were then compared between low and high expression groups. Interestingly, only high *PRKD1* expression was associated with a lower metastasis-free survival independently of the BC subtype (p=0.0083; Figure 1C), whereas *PRKD2* or *PRKD3* expressions did not significantly correlate with prognosis (Supplementary Figure 2). High *PRKD1* mRNA levels were also significantly associated with a low SBR histological grade, ER- status and ERBB2+ status in the entire cohort (Supplementary Table 1).

We next assessed the prognostic value of *PRKD1* expression in the different BC subtypes (TNBC, HR-/ERBB2+, HR+/ERBB2- and HR+/ERBB2+) and found that high *PRKD1* mRNA levels were associated with a lower MFS in TNBC (p=0.010; Figure 1D) but not in HR-/ERBB2+, HR+/ERBB2- or HR+/ERBB2+ tumors (Supplementary Figure 2). Importantly, high *PRKD1* expression was also significantly associated with a lower overall survival in TNBC (p=0.022; Supplementary Figure 3). High *PRKD1* mRNA levels did not correlate with clinical, pathological or biological parameters in TNBC (Supplementary Table 2).

In order to validate our findings in an independent cohort, we next examined the prognostic value of *PRKD1* expression in a publicly available breast cancer database (KMPLOT; http://kmplot.com) [15]. This database contains gene expression data and distant metastasis-free survival information for 1747 breast cancer patients. As expected, high *PRKD1* mRNA levels were associated with a lower metastasis-free survival in both the entire BC population and the TNBC subgroup in the validation cohort (Supplementary Figure 4).

To determine whether *PRKD1* could be an independent prognostic factor, we next performed a multivariate analysis in both the entire BC population and the TNBC subgroup in our cohort. We tested the influence of *PRKD1* mRNA levels on MFS, together with the histological grade, lymph-node status, pathological size, and progesterone receptor status (Table 1). We found that lymph node invasion (p=0.0000009), high pathological size (p=0.002), high histological grade (p=0.03) and high *PRKD1* expression (p=0.003) were significantly associated with a poorer prognosis in the whole breast cancer population (Table 2A). Most interestingly, high *PRKD1* expression was the only factor predicting MFS in TNBC (p=0.008) (Table 2B).

**Table 2A:** Multivariate analysis of the influence of *PRKD1* expression on MFS in the series of 527 breast tumors

Characteristics		HR^a^	95% CI^b^	*p*-value^c^
*Lymph node status*	0	1		
	1-3	1.64	1.34-1.99	**0.0000009**
	>3	2.67	1.81-3.96	
*Pathological size*	≤25mm	1		
	>25mm	1.58	1.18-2.11	**0.002**
*SBR histological grade*	I	1		
	II	1.29	1.03-1.61	**0.03**
	III	1.66	1.06-2.60	
*PR status*	positive	1		
	negative	1.3	0.97-1.75	0.08 (NS)
*PRKD1 expression*	Low	1	1.16-2.01	**0.003**
	High	1.52		

^a^: Hazard ratio.

^b^: 95% Confidential interval.

^c^: Multivariate COX analysis.

**Table 2B:** Multivariate analysis of the influence of *PRKD*1 expression on MFS in the series of 102 triple-negative breast tumors

Characteristics		HR^a^	95% CI^b^	*p*-value^c^
*Lymph node status*	0	1	0.79-1.92 0.62-3.69	0.37 (NS)
	1-3	1.23		
	>3	1.51		
*Pathological size*	≤25mm	1		
	>25mm	1.75	0.87-3.53	0.12 (NS)
	1-3	1.23	0.79-1.92	
	>3	1.51	0.62-3.69	0.37 (NS)
*SBR histological grade*	I	1		
	II	1.2	0.69-2.09	0.51 (NS)
	III	1.45	0.48-4.39	
*PRKD1 expression*	Low	1		
	High	2.54	1.28-5.03	**0.008**

^a^: Hazard ratio.

^b^: 95% Confidential interval.

^c^: Multivariate COX analysis.

Together, these results show that *PRKD1*, *PRKD2* and *PRKD3* are expressed in breast cancer and that *PRKD*1 mRNA expression is an independent prognostic factor in the entire BC population and in the TNBC subpopulation. The poorer prognosis of TNBC expressing high *PRKD1* levels suggests that PKD1 plays a role in the biology of TN breast tumors and could represent a therapeutic target for their treatment.

### Effect of PKD1 inhibition in TNBC cells

To determine whether PKD1 could be a relevant therapeutic target in TNBC, we next assessed the effect of PKD1 inactivation in cellular assays.

Since no selective PKD1 pharmacological inhibitor has been reported to date, we first screened a large panel of compounds to identify those capable of inhibiting PKD1 kinase activity in a biochemical assay. Forty-one molecules were found to inhibit PKD1 activity *in vitro* with an IC50 lower than 480 nM (range 9 – 480 nM).

In a previous work, we have shown that PKD1 overexpression confers estrogen independence in MCF7 breast cancer cells [7]. Interestingly, MCF7 parental cells are not able to grow on a semi-solid medium in the absence of estrogens whereas MCF7-PKD1 cells form colonies under the same conditions [7]. Thus, the effect of the 41 potential PKD1 inhibitors was assessed in MCF7-PKD1 cells grown on methylcellulose in the absence of estrogens. The majority of molecules (32 out of 41) were able to inhibit estrogen-independent clonogenicity of MCF7-PKD1 cells with an IC50 below 10 μM (Supplementary Table 3). Two compounds were selected for further experiments: AB9539, which showed the lowest IC50 (0.23 μM) and AB9275 which demonstrated a higher IC50 (1.27 μM) but possesses a good bioavailability (about 20%).

**Figure 2:**
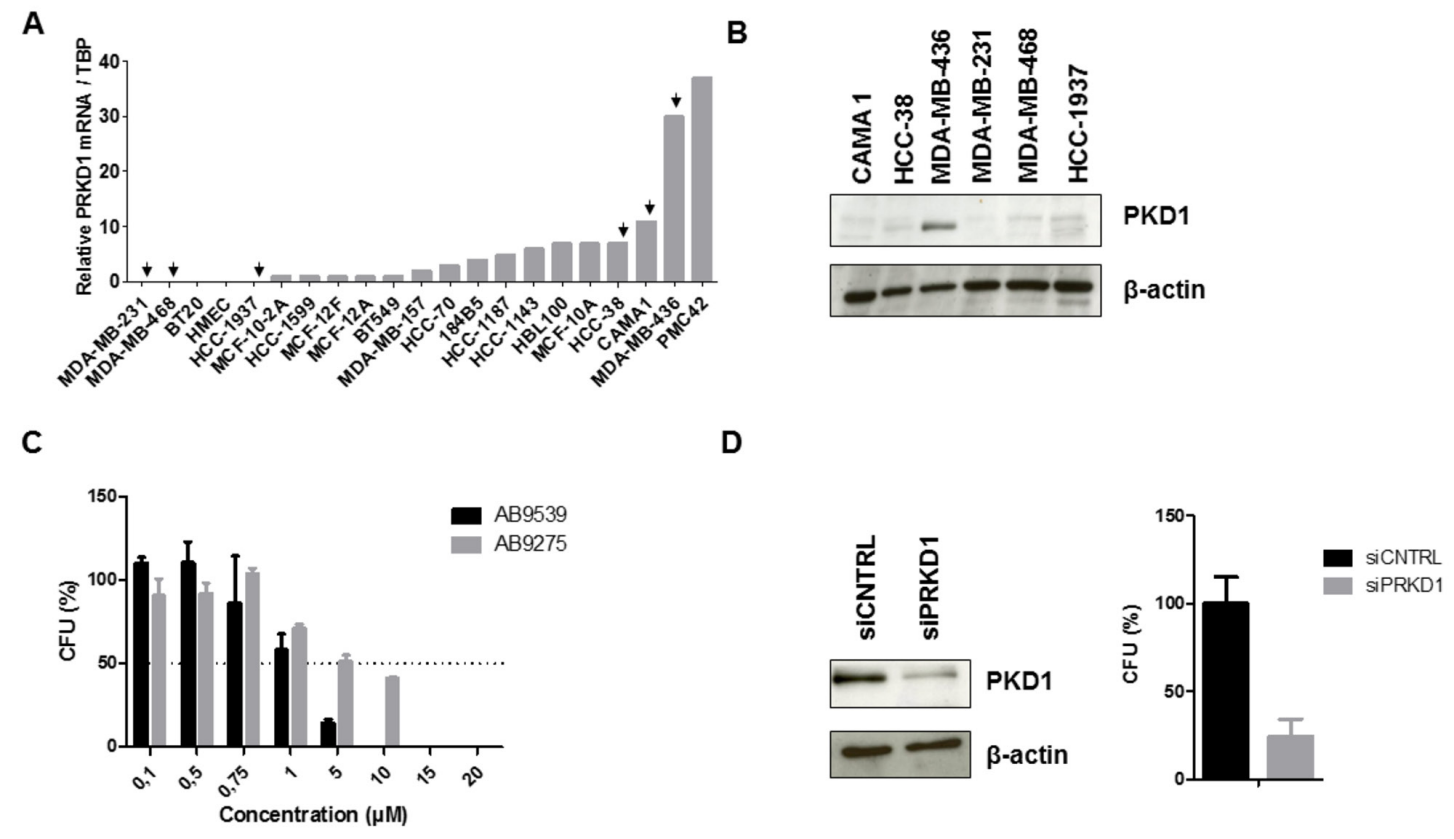
Effect of PKD1 inhibition in TNBC cells **(A)** *PRKD1* mRNA levels in 21 TNBC cell lines. *PRKD1* mRNA expression was analyzed by RT-qPCR and normalized to that of the *TBP* control gene. **(B)** Western-blot analysis of PKD1 protein expression in six TNBC cell lines. β-actin was used as a loading control. **(C)** Effect of PKD1 pharmacological inhibitors on clonogenicity of MDA-MB-436 cells. CFU, colony forming unit. Untreated cells were used as a reference (100%). Mean values ± SEM from two independent experiments are shown. **(D)** Effect of siRNA-mediated PKD1 knockdown on MDA-MB-436 clonogenicity. MDA-MB-436 cells were transfected with 50 nM non-targeting (siCNTRL) or PKD1-targeting (si*PRKD*1) siRNAS during 48 hours. Left: western-blot showing the efficiency of PKD1 silencing. β-actin was used as a loading control. Right: colony formation was evaluated after two weeks. Cells transfected with non-targeting siRNAs were used as a reference (100%). Mean values ± SEM from two independent experiments are shown.

To evaluate the effect of PKD1 pharmacological inhibition in TNBC cells, *PRKD1* transcript levels were first analyzed by quantitative RT-PCR in a series of 21 TNBC cell lines. We found that *PRKD1* was expressed at the mRNA level in about half of the TNBC cell lines (Figure 2A). PKD1 protein expression was then analyzed by western blot in three cell lines showing *PRKD1* mRNA expression (MDA-MB-436, CAMA-1, HCC-38) and in three cell lines in which *PRKD1* transcript was not detected (MDA-MB-231, MDA-MB-468, HCC-1937). PKD1 protein expression was observed only in MDA-MB-436 cells (Figure 2B), which also expressed high levels of *PRKD1* mRNA. Thus, this cell line was selected to further study the effect of PKD1 inhibition. MDA-MB-436 cells were treated with different concentrations of the AB9539 or AB9275 compounds and colonies were counted after two weeks. Interestingly, both molecules were able to reduce colony formation, with an IC50 of 2.0 μM and 4.1 μM for AB9539 and AB9275, respectively (Figure 2C). Importantly, PKD1 silencing also resulted in a marked inhibition of clonogenicity in MDA-MB-436 cells (Figure 2D).

In conclusion, we identified two novel PKD1 inhibitors and demonstrated that both pharmacological inhibition and siRNA-mediated depletion of PKD1 reduces the ability of PKD1-expressing TNBC cells to form colonies.

### Antitumor activity of the AB9275 PKD1 inhibitor *in vivo*

To examine whether pharmacological inhibition of PKD1 could inhibit tumor growth *in vivo*, we decided to evaluate the antitumor activity of the AB9275 molecule in a TNBC patient-derived xenograft (PDX) model. AB9275 was preferred over AB9539 because of its better bioavailability (20% versus less than 5%).

**Figure 3:**
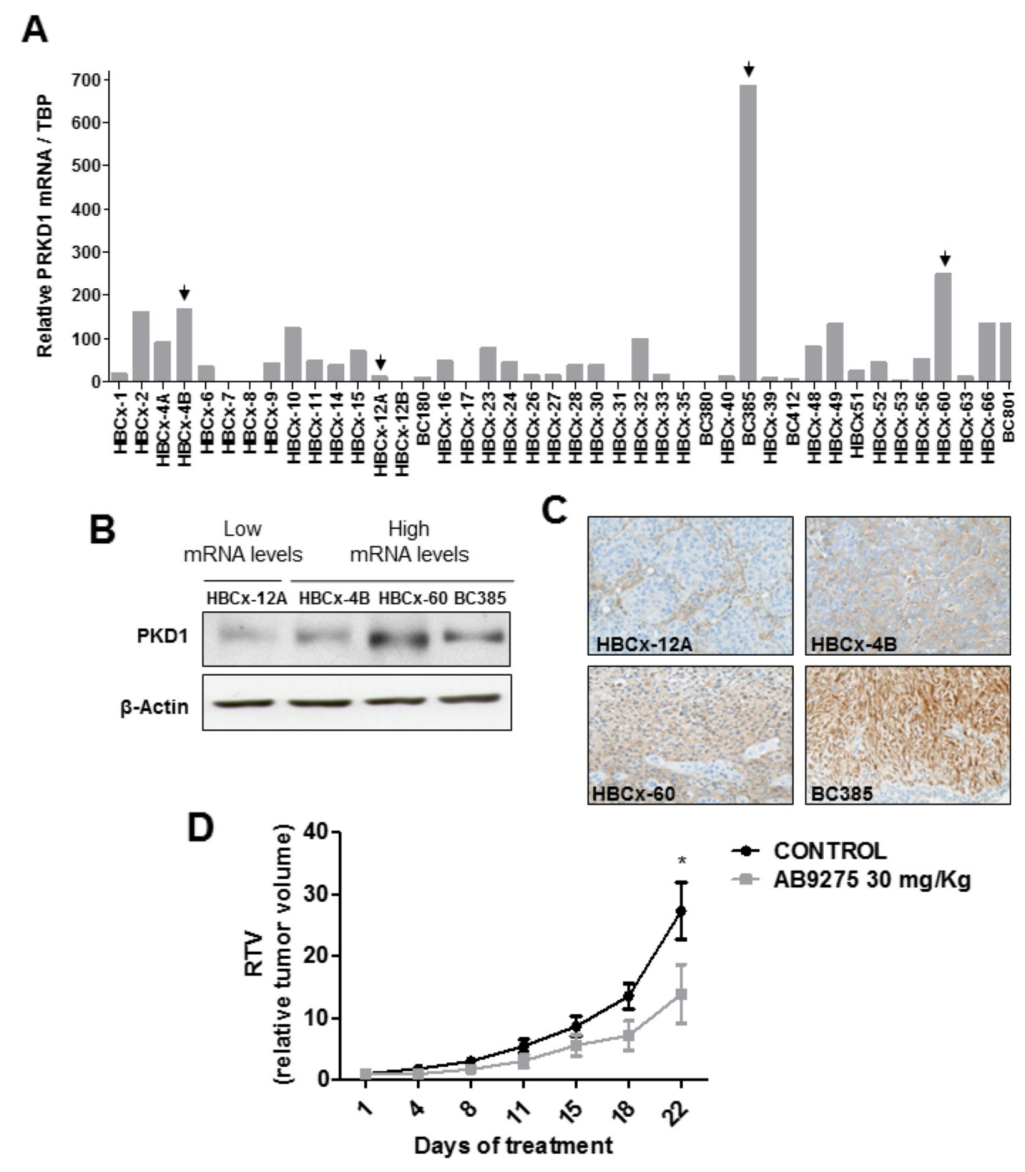
*In vivo* antitumor activity of the AB9275 PKD1 inhibitor against a TNBC PDX **(A)** *PRKD1* mRNA levels in 41 TNBC PDXs. *PRKD1* mRNA expression was analyzed by RT-qPCR and normalized to that of the *TBP* control gene. **(B)** Western-blot analysis of PKD1 protein expression in four PDXs expressing high or intermediate *PRKD1* mRNA levels (HBCx-4A, HBCx-60, BC385) and low *PRKD1* mRNA levels (HBCx-12A). β-actin was used as a loading control. **(C)** Immunohistochemical analysis of PKD1 protein expression in the HBCx-4A, HBCx-60, BC385, and HBCx-12A tumors. Original magnification x200. **(D)** Effect of AB9275 on tumor growth in the HBCx-60 PDX model. Mice bearing HBCx-60 tumors were treated *per os* once daily during 22 days with 30 mg/kg AB9275 (n=7) or water (n=8). Mean RTV±SEM are shown. ^*^, P < 0.05; Wilcoxon-Mann-Whitney test.

In order to select a relevant *in vivo* model, PKD1 expression was analyzed in a panel of 41 TNBC PDXs. *PRKD1* mRNA expression was detected by quantitative RT-PCR in about half of the PDXs (Figure 3A). The three xenografts showing the highest *PRKD1* mRNA levels (BC385, HBCx-60 and HBCx-4B) were selected for further experiments. The HBCx-12A model, expressing low *PRKD1* mRNA levels, was selected as a negative control. High PKD1 protein expression was confirmed by western blot and immunohistochemistry in BC385, HBCx-60 and HBCx-4B (Figure 3A–3C). Conversely, PKD1 protein expression was low in the HBCx-12A model (Figure 3A–3C). The HBCx-60 PDX model, which expresses the highest levels of PKD1 protein (Figure 3B–3C), was selected for *in vivo* evaluation of AB9275 activity.

Athymic mice bearing HBCx-60 xenografts were randomized into control and treatment groups and received either vehicle or AB9275 at 30 mg/kg, once daily during 22 days. The drug was well tolerated since body weight remained stable among treated animals and no toxic death was observed. Importantly, treatment with AB9275 was potent against the HBCx-60 model with a tumor growth inhibition (TGI) of 49% at the end of the treatment (p<0.05; Figure 3D).

These data demonstrate that pharmacological inhibition of PKD1 is able to inhibit tumor growth in a PKD1-expressing TNBC model *in vivo*.

## DISCUSSION

We previously reported that overexpression of PKD1 confers estrogen independence to ER+ breast cancer cells and is associated with a poorer prognosis in ER+ tamoxifen-treated breast tumors [6]. This prompted us to determine whether PKD1 could be a potential prognostic factor and/or a therapeutic target in breast cancer. We showed that among the three members of the PKD family, only PKD1 is an independent prognostic factor in our entire breast cancer cohort. Indeed, *PRKD2* and *PRKD3* expression levels had no influence on MFS in the same series of tumors. It has been previously shown that PKD1 is down-regulated in breast cancer as compared to normal breast tissue [16, 17]. However, the association between PKD1 expression and outcome has never been examined. In our cohort, we did observe that PKD1 is down-regulated in primary breast tumors as compared to normal breast tissue (data not shown) and we also showed that high *PRKD1* mRNA levels are predictive of a poorer prognosis in both the entire cohort and the TNBC subgroup. Borges et al. reported that high *PRKD3* expression is a poor prognostic factor in ER- breast tumors [14]. However, we were not able to reach the same conclusions in our series of tumors (Supplementary Table 1).

The biological role of PKD1 in breast cancer is still unclear but we have previously demonstrated that it can drive estrogen independence in ER+ BC cells [6]. Most interestingly, high *PRKD1* expression is a poor prognostic factor in ER+ tamoxifen-treated breast tumors, suggesting that PKD1 participates to endocrine therapy resistance in the clinics [6]. In the present study, we found that *PRKD1* expression is associated with a poor prognosis specifically in TNBC, *i.e.* in another hormone-independent BC subtype. Together, these data indicate that PKD1 is likely to play a specific role in estrogen-independent breast tumors.

Several potential PKD1 inhibitors have been reported in the literature. They were shown to inhibit PKD1 *in vitro* with IC50s comprised between 1 and 200 nM but none of them is specific for PKD1 [18–25]. Indeed, the CID755673, CRT5 and CRT0066101 compounds similarly inhibit PKD1 and PKD3 while the Gö6976 molecule (which has been used extensively to inhibit PKD1) also inhibits PKCα and PKCβ (Supplementary Figure 5A) [18–20, 23, 25]. Moreover, a major limitation of PKD inhibitors is that they exhibit a limited bioavailability [26]. Thus, we identified for the first time a specific PKD1 inhibitor which can be administered *in vivo*. Indeed, the AB9275 molecule did not inhibit PKD3, PKCα or PKCβ *in vitro* at concentrations inhibiting PKD1, whereas CRT0066101 was more potent against PKD3 and Gö6976 strongly inhibited PKCα and PKCβ in the same assays (Supplementary Figure 5A). AB9275 also showed a good selectivity profile in a competition assay against a broad panel of kinases (Supplementary Figure 5B). In addition, the molecule showed no toxicity in mice at the dose of 30 mg/kg/day and demonstrated a good bioavailability (about 20%).

Pharmacological inhibition or siRNA-mediated depletion of PKD1 has previously shown cytotoxicity in melanoma, breast cancer, prostate cancer and pancreatic cancer cells [11, 13, 19, 25, 27]. The CRT0066101 compound was also able to inhibit tumor growth of MCF7 chemoresistant xenografts *in vivo* [13]. In the present study, we show that TNBC cells are also sensitive to PKD1 inhibition or PKD1 knockdown. In addition, we demonstrate that PKD1 inhibition reduces tumor growth *in vivo* in a TNBC PDX model. Together, these results suggest that PKD1 could be a relevant therapeutic target in TNBC.

Interestingly, the AB9275 PKD1 inhibitor demonstrated antitumor activity *in vivo* in spite of its moderate ability to inhibit clonogenicity of MFC7-PKD1 and MDA-MB-436 cells (IC50 of 1.3 and 4.1 μM, respectively). Conversely, the AB9539 molecule was more potent in cellular assays (IC50 of 0.2 and 2.0 μM against MCF7-PKD1 and MDA-MB-436 cells, respectively) but could not be evaluated *in vivo* because of its low bioavailability. Improving the pharmacokinetic and pharmacodynamic properties of our current inhibitors should thus generate much more potent molecules, which could be developed for the treatment of TNBC, as well as other malignancies.

In conclusion, we showed for the first time that PKD1 is an independent prognostic factor and a promising therapeutic target in TNBC. The development of potent PKD1 inhibitors could provide a novel treatment option for TNBC patients.

## MATERIALS AND METHODS

### Patients

Primary breast tumor samples were obtained from 527 women treated at Institut Curie - Hôpital René Huguenin (Saint-Cloud, France) between 1978 and 2008. All patients treated at Institut Curie before 2007 were informed that their tumor samples might be used for scientific purposes and had the opportunity to decline. Since 2007, patients treated at Institut Curie have given their approval by signing an informed consent. This study was approved by the local ethics committee (Breast Group of Institut Curie - René Huguenin Hospital). The samples were immediately stored in liquid nitrogen until RNA extraction. A tumor sample was considered suitable for this study if the proportion of tumor cells exceeded 70%. All patients (mean age 60.9 years, range 29 – 91 years) met the following criteria: primary unilateral non metastatic breast carcinoma for which complete clinicopathological data and follow-up were available; no radiotherapy or chemotherapy before surgery; and full follow-up at Institut Curie - Hôpital René Huguenin. Adjuvant therapy was administered to 367 patients, consisting of chemotherapy alone in 95, hormone therapy alone in 177, and both treatments in 95 patients. Estrogen receptor (ER), progesterone receptor (PR), and human epidermal growth factor receptor 2 (ERBB2) statuses were determined at the protein level by biochemical methods (Dextran-coated charcoal method, enzyme immunoassay or immunohistochemistry) and confirmed by real-time quantitative RT-PCR [28, 29]. The population was divided into four groups according to HR (ER and PR) and ERBB2 statuses as follows: two luminal subtypes [HR+ (ERα+ or PR+)/ERBB2+ (n=58)] and [HR+ (ERα+ or PR+)/ERBB2- (n=295)]; an ERBB2+ subtype [HR- (ERα- and PR-)/ERBB2+ (n=72)] and a triple-negative subtype[HR- (ERα- and PR-)/ERBB2- (n=102)]. Within a median follow-up of 9.4 years (range 1 month to 33.2 years), 210 patients developed distant metastasis.

### RNA extraction

Total RNA was extracted from breast tumor samples by using acid-phenol guanidium as previously described [30]. RNA quality was determined by electrophoresis through agarose gels, staining with ethidium bromide, and visualization of the 18S and 28S RNA bands under ultraviolet light.

### Real-time RT-PCR

Quantitative values were obtained from the cycle number (Ct value) at which the increase in the fluorescence signal associated with exponential growth of PCR products started to be detected by the laser detector of the ABI Prism 7900 Sequence Detection System (Perkin-Elmer Applied Biosystems, Foster City, CA), using PE Biosystems analysis software according to the manufacturer’s manuals. The TBP gene (Genbank accession NM_003194) encoding the TATA box-binding protein (a component of the DNA-binding protein complex TFIID) was quantified as an endogenous RNA control, and each sample was normalized on the basis of its TBP content [28]. Results, expressed as N-fold differences in target gene expression relative to the TBP gene and termed “Ntarget”, were determined as Ntarget = 2ΔCtsample, where the ΔCt value of the sample was determined by subtracting the average Ct value of the target gene from the average Ct value of the TBP gene. The smallest amount of mRNA that was detectible (ΔCt=35) was used as a reference (basal mRNA level=1) to normalize the data for cell lines and xenograft samples. For primary tumors, the median target gene value of normal breast tissues (ten samples) was used as a reference to normalize the data. All ratios were then multiplied by 10 (reference mRNA level=10). Primers’ sequences are available on request. The conditions of cDNA synthesis and PCR have been described previously [28].

### Cell culture

Breast cancer cell lines were obtained from the American Type Culture Collection (ATCC, Manassas, VA, USA). PKD1 overexpression by stable transfection has been previously described [7]. MCF7-PKD1 cells were cultured in DMEM-Glutamax medium, supplemented with 10% fetal bovine serum (FBS) and 100 units/mL penicillin and 100 mg/mL streptomycin (P/S) (Invitrogen Life Technologies, Cergy-Pontoise, France). 1 mg/mL G418 (Calbiochem, Darmstadt, Germany) was added for the culture of stably transfected MCF7-PKD1 cells. MDA-MB-436 cells were cultured in RPMI 1640 medium supplemented with 10% FBS P/S. HCC38 cells were cultured in RPMI 1640 medium supplemented with 10% FBS, P/S and 1% sodium pyruvate. CAMA-1 cells were cultured in MEM (eagle) medium supplemented with 10% FBS and P/S.

### Western blot analysis

Cells were lysed for 20 min at 4°C in 50 mM Tris–HCl pH 7.4, 150 mM NaCl, 1 mM EDTA, 100 mM sodium fluoride, 10 mM tetra-sodium diphosphate decahydrate, 2 mM sodium orthovanadate, 1 mM PMSF, 10 μg/mL aprotinin and 1% Nonidet P-40. Lysates were clarified by centrifugation at 14,000 rpm for 10 min at 4°C. 30–80 μg of total proteins were separated by SDS-PAGE and transferred onto nitrocellulose membranes. These were incubated with specific antibodies and revealed by enhanced chemiluminescence (Amersham, GE Healthcare, UK).

The following antibodies were used at the indicated dilutions: anti-PKD1: 1/1000 (HPA029834; Sigma-Aldrich, Saint Quentin Fallavier, France), anti-β-actin: 1/5000 (A5441; Sigma-Aldrich, Saint Quentin Fallavier, France), horseradish peroxidase-conjugated goat anti-rabbit IgG: 1/2000 (P0448, Dako, Glostrup, Denmark) and horseradish peroxidase-conjugated goat anti-mouse IgG: 1/5000 (610-1302, Rockland, Gilbertsville, PA, USA).

### Anchorage-independent growth assay

10,000 MCF7-PKD1 cells were suspended in 2.5 mL of methylcellulose (0.8%) prepared in estrogen-free medium containing or not different concentrations of PKD1 inhibitors or dimethylsulfoxide (DMSO). Cells were plated in uncoated 35-mm culture dishes and incubated for three weeks. Then, macroscopic colonies were counted.

### Colony formation assay

8,000 cells were seeded in 6-well plates with complete medium containing or not different concentrations of PKD1 inhibitors or DMSO. Macroscopic colonies were counted after two weeks.

### siRNA transfection

50,000 cells were transfected with 50 nM *PRKD*1-targeting or nontargeting siRNAs (L-005028-00-0005, smartpool siRNA targeting *PRKD1*, Dharmacon, Colorado, USA), and 48h after transfection, 8 000 cells were plated in 6-well plates for colony formation assay.

### *In vivo* experiments

*In vivo* experiments were performed on female Swiss nude mice purchased from Charles River (Saint-Germain-sur-l’Arbresle, France). Mice care and housing were conformed to the institutional guidelines as put forth by the French Ethical Committee. Human TNBC xenograft models were established as previously described [31, 32]. A toxicity study was first performed on mice bearing human BC xenografts which received 20 or 30 mg/kg of AB9275 *per os* once daily during 22 days. As no toxicity was observed, the dose of 30 mg/kg was selected for the next experiments. For the evaluation of AB9275 antitumor activity, the mice received the drug (treated group) or water (control group) *per os* once daily during 22 days. Tumor growth was evaluated with a caliper twice a week. Tumor growth inhibition (TGI) of treated tumors versus controls was calculated as the ratio of the mean relative tumor volume (RTV) in the treated group to the mean RTV in the control group at the same time.

### Immunohistochemistry

Paraffin-embedded breast tumors samples, obtained at the time of initial diagnosis, were retrieved from the archives of the Department of Biopathology at René Huguenin Hospital. Sections of 3 μm in thickness were cut with a microtome from the paraffin-embedded tissue blocks of normal breast tissue, pre-invasive lesions and IBCs (invasive breast cancer). Tissue sections were dewaxed and rehydrated through a series of xylene and ethanol washes. Immunostaining was performed on a Dako automated system. Primary antibody against PKD1 (Cell signaling, Danvers, MA) was incubated overnight at 4°C (dilution 1/100).

Patient-derived xenografts were fixed in 10% neutral buffered formalin and embedded in paraffin. Tissue sections were immunostained in a Discovery XT Platform (Ventana Medical System, Tucson, Arizona, USA, part of Roche Diagnostics) using EDTA buffer pH 8.0 (CC1, Ventana Medical System) for antigen retrieval. Primary antibody against PKD1 (Cell signaling, Danvers, MA) was incubated during 30 min at 37°C (dilution 1/100). After incubation with anti-rabbit secondary antibodies, slides were covered with the chromogenic substrate diaminobenzidine (ChromoMap Kit with Anti rabbit OmniMap, Ventana Medical System) and counterstained with hematoxylin.

### Bioinformatics

KM Plotter data were obtained using the current release of Kaplan Meier Plotter (www.kmplot.com; [15]; 2017 version, n=1809), interrogating the database using Affymetrix ID “205880_at” for distant metastasis-free survival (no follow-up threshold). The best cutoff value was automatically selected and biased arrays were excluded from the analysis.

### Statistical analysis

Relationships between mRNA levels and clinical parameters were identified using the chi-square test.

Metastasis-free survival (MFS) was determined as the interval between initial diagnosis and detection of the first metastasis. Survival distributions were estimated by the Kaplan-Meier method and the significance of differences between survival rates were ascertained with the log-rank test. The cox proportional hazards regression model was used to assess prognostic significance and the results are expressed as hazard ratios and 95% confidence intervals.

The Wilcoxon-Mann-Whitney test was used to compare individual RTVs at the end of the experiment between treated and control groups.

## SUPPLEMENTARY MATERIALS FIGURES AND TABLES









## Abbreviations

BC: Breast cancer
TNBC: Triple-negative breast cancer
MFS: Metastasis-free survival
HR: Hormone receptors
ER: Estrogen receptor
RTV: Relative tumor volume
