# Supplementary material for: PKD1 is a potential biomarker and therapeutic target in triple-negative breast cancer
Source: Oncotarget. 2018 May 1;9(33):23208–19. doi: 10.18632/oncotarget.25292 (PMC5955414; doi:10.18632/oncotarget.25292)
Supplement: Supplementary file 1 [file oncotarget-09-23208-s001.pdf]

PKD1 is a potential biomarker and therapeutic target in triple-negative breast cancer

SUPPLEMENTARY MATERIALS

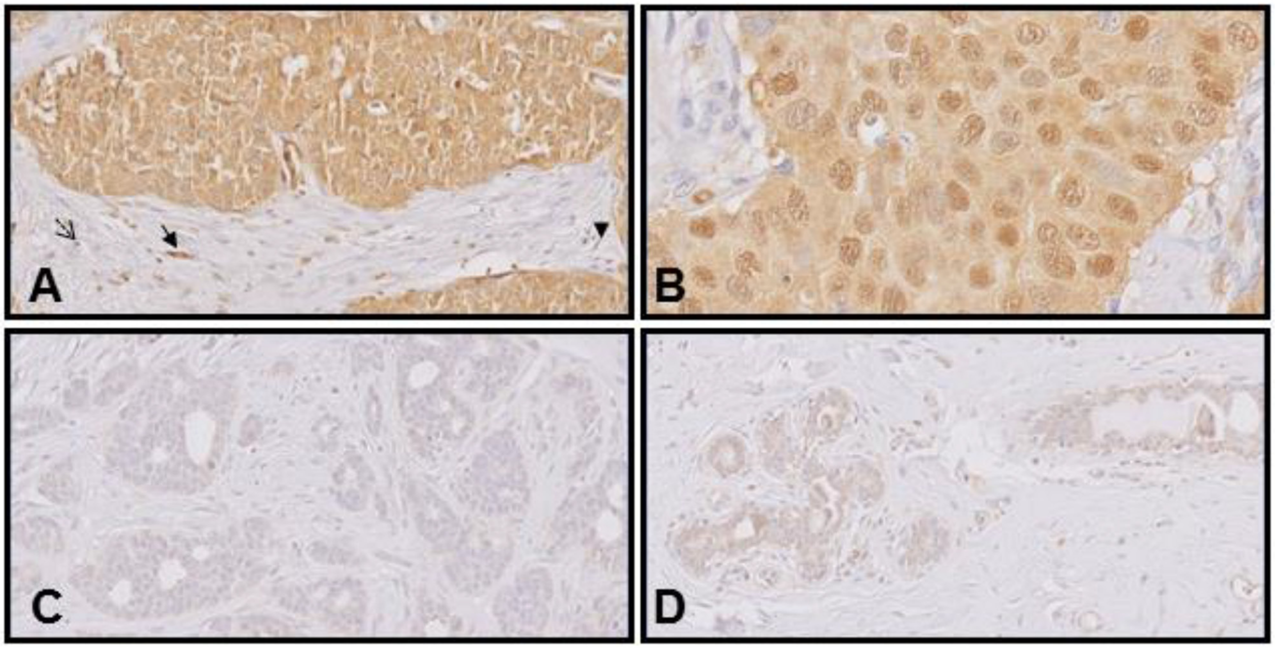

Supplementary Figure 1: Immunohistochemical analysis of PKD1 protein expression in primary breast tumors **(A-C)** and normal mammary tissue **(D)**. A) Breast tumor sample showing a high PKD1 expression in tumor cells and in cells from the tumor microenvironment (arrow: fibroblast; dotted arrow: mononuclear immune cell; arrowhead: endocyte). B) Breast tumor sample showing both cytoplasmic and nuclear PKD1 staining. C) Breast tumor sample showing a low PKD1 expression. Original magnification x400 (A, C and D); x1000 (B).

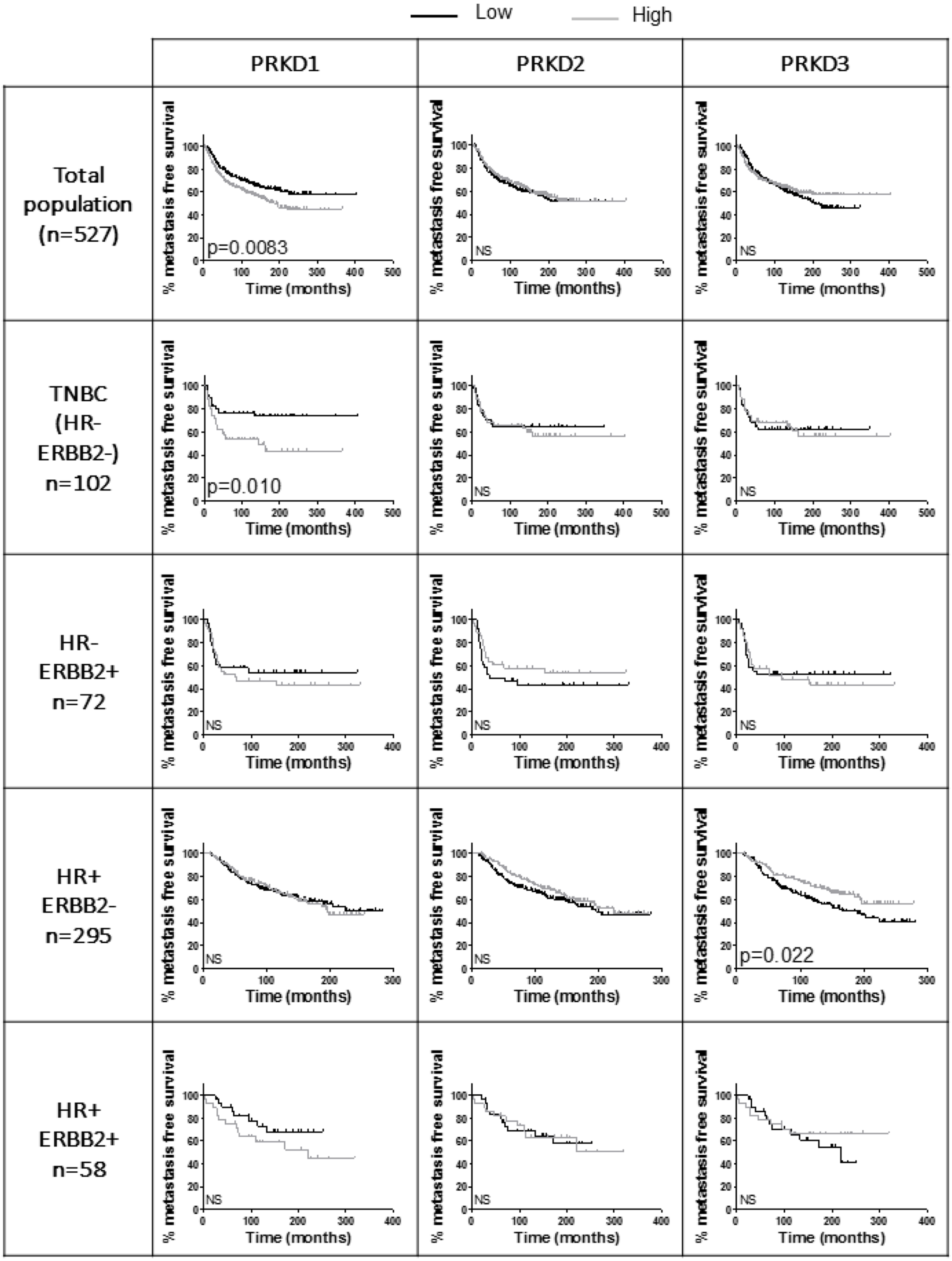

Supplementary Figure 2: Kaplan-Meier analysis of metastasis-free survival according to *PRKD1*, *PRKD2* and *PRKD3* expressions in the entire BC cohort and in the four BC subtypes.

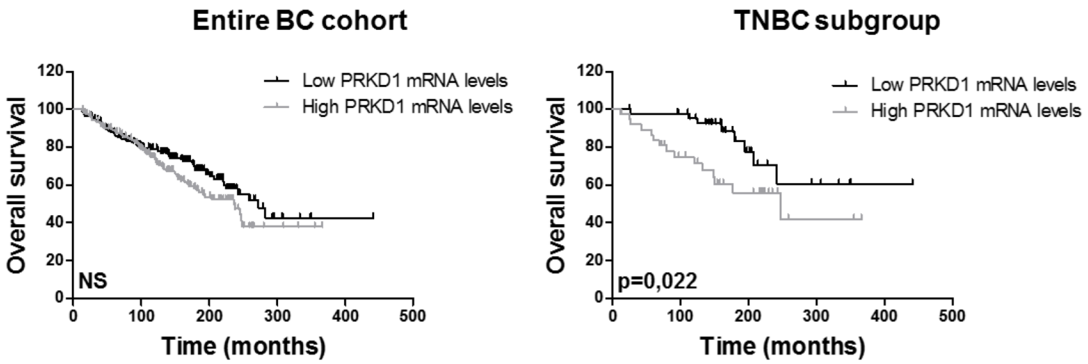

Supplementary Figure 3: Kaplan-Meier analysis of overall survival according to *PRKD1* expression in the BC cohort (n=446) and the TNBC subgroup (n=82).

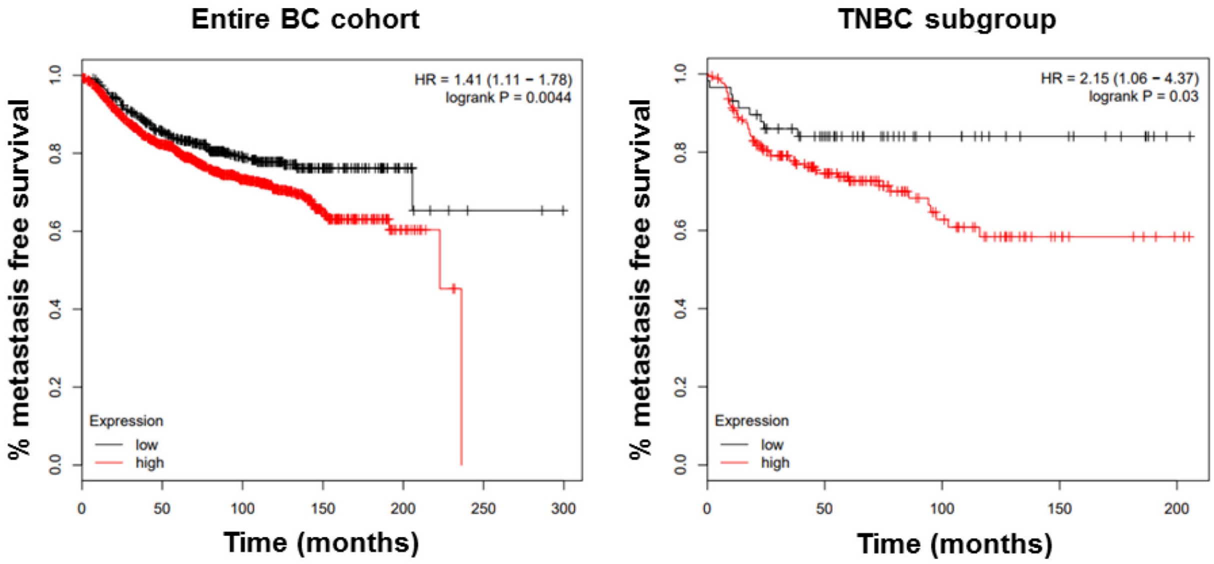

Supplementary Figure 4: Kaplan-Meier analysis of distant metastasis-free survival according to *PRKD1* expression in the entire BC population (n=1746) and the TNBC subgroup (n=232) in a publicly available breast cancer database (www.kmplot.com). The best cutoff was automatically selected.

A

PKD1 inhibitors	IC50 (nM)			
	PKD1	PKD3	PKCα	PKCβ
**AB9275**	37	175	20000	20000
**CRT0066101**	4,5	1,6	no inhibition[1]	no inhibition[1]
**Gö6976**	25	630	20	45
**CID 755673**	182 [2]	227 [2]	10000 [2]	10000 [2]
**CRT5**	1 [3]	1.5 [3]	no inhibition[3]	no inhibition[3]

[1] Harikumar et al., 2010

[2] Sharlow et al., 2008

[3] Evan et al., 2010

B

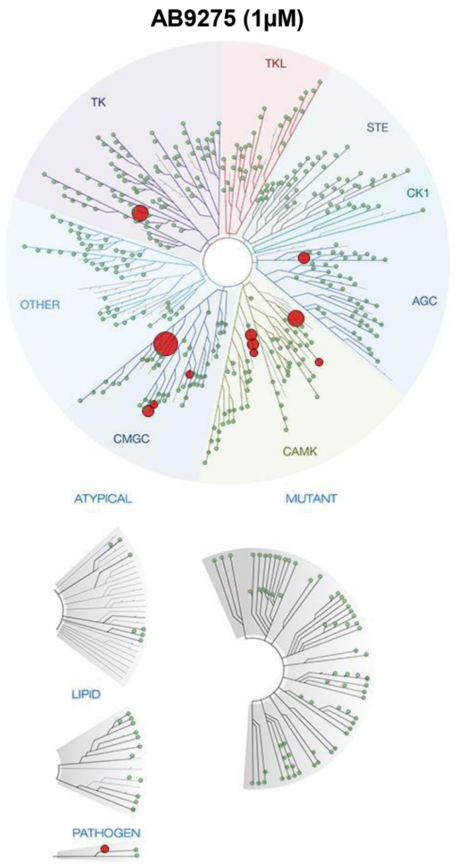

Supplementary Figure 5: Specificity of the AB9275 PKD1 inhibitor. **(A)** Comparison of *in vitro* kinase inhibitory activities between AB9275 and different PKD1 inhibitors. Mean IC50s were obtained from two independent experiments for AB9275, Gö6976 and CRT0066101. Other IC50s were obtained from the literature (as indicated). **(B)** Kinome selectivity profile of the AB9275 compound. The selectivity of AB9275 towards a panel of 442 kinases was determined using the KINOMEscan® competition assay (DiscoveRx, Fremont CA). AB9275 was screened at 1 μM. Kinases found to bind the compound are marked with red circles and larger circles indicate higher-affinity binding.

Supplementary Table 1: Relationship between *PRKD1* mRNA levels and classical clinical/biological parameters in the entire BC cohort.

See Supplementary File 1

Supplementary Table 2: Relationship between *PRKD1* mRNA levels and classical clinical/biological parameters in the TNBC subgroup.

See Supplementary File 2

Supplementary Table 3: Inhibition of estrogen-independent clonogenicity of MCF7-PKD1 cells by the 41 PKD1 inhibitors.

See Supplementary File 3
