# Supplementary material for: PKD1 is a potential biomarker and therapeutic target in triple-negative breast cancer
Source: Oncotarget. 2018 May 1;9(33):23208–19. doi: 10.18632/oncotarget.25292 (PMC5955414; doi:10.18632/oncotarget.25292)
Supplement: Supplementary file 2 [file oncotarget-09-23208-s002.docx]

**Supplemental table 1: Relationship between *PRKD1* mRNA levels and classical clinical/biological parameters in the entire BC cohort**

|  | Number of patients (%) | | | |
| --- | --- | --- | --- | --- |
|  | Total population (%) | Low *PRKD1* mRNA levels | High *PRKD1* mRNA level | *p* a |
| *Total* | 527 (100) | 272 (51.6) | 255 (48.3) |  |
| *Age*  ≤50  >50 | 125 (23.7)  402 (76.3) | 56 (44.8)  216 (53.7) | 69 (55.2)  186 (46.3) | 0.081 (NS) |
| *SBR histological grade* b,c  I  II  III | 60 (11.7)  241 (47.1)  211 (41.2) | 22 (36.7)  126 (52.3)  118 (55.9) | 38 (63.3)  115 (47.7)  93 (44.1) | **0.030** |
| *Lymph node status* d  0  1-3  >3 | 159 (30.5)  250 (47.9)  113 (21.6) | 89 (55.9)  128 (51.2)  51 (45.1) | 70 (44.1)  122 (48.8)  62 (54.8) | 0.21 (NS) |
| *Pathological size* e  ≤25mm  >25mm | 248 (48.0)  269 (52.0) | 122 (49.2)  145 (53.9) | 126 (50.8)  124 (46.1) | 0.28 (NS) |
| *ER status*  Negative  Positive | 181 (34.3)  346 (65.7) | 81 (44.7)  191 (55.2) | 100 (55.3)  155 (44.8) | **0.023** |
| *PR status*  Negative  Positive | 255 (48.4)  272 (51.6) | 130 (51.0)  142 (52.2) | 125 (49.0)  130 (47.8) | 0.78 (NS) |
| *ERBB2 status*  Negative  Positive | 397 (75.3)  130 (24.7) | 215 (54.2)  57 (43.8) | 182 (45.8)  73 (56.2) | **0.041** |
| *Molecular subtypes*  RH- ERBB2-  RH- ERBB2+  RH+ ERBB2-  RH+ ERBB2+ | 102 (19.4)  72 (13.7)  295 (56.0)  58 (11.0) | 53 (52.0)  28 (38.9)  162 (54.9)  29 (50.0) | 49 (48.0)  44 (61.1)  133 (45.1)  29 (50.0) | 0.11 (NS) |
| *PIK3CA mutation status f*  wild type  mutated | 299 (67.0)  147 (33.0) | 167 (55.9)  76 (51.7) | 132 (44.1)  71 (48.3) | 0.41 (NS) |
| *KI67 mRNA expression g, h*  Median | 12.5 (0.80-313) | 13.0 (0.8-313.0) | 11.6 (0.9-106.7) | 0.066 (NS) |
| *Histological subtypes i* |  |  |  |  |
| Apocrine | 2 (0.45) | 0 | 2 (100.0) | 0.29 (NS) |
| Colloid | 4 (0.90) | 1 (25.0) | 3 (75.0) |  |
| Ductal | 398 (89.6) | 219 (55.0) | 179 (45.0) |  |
| Lobular | 28 (6.3) | 14 (50.0) | 14 (50.0) |  |
| Medullary | 4 (0.90) | 3 (75.0) | 1 (25.0) |  |
| Metaplastic | 1 (0.23) | 1 (100.0) | 0 |  |
| Mixed | 5 (1.13) | 1 (20.0) | 4 (80.0) |  |
| Papillary | 1 (0.23) | 1 (100.0) | 0 |  |
| Tubular | 1 (0.23) | 1 (100.0) | 0 |  |

a χ2Test

b Scarff Bloom Richardson classification.

c Information available for 512 patients.

d Information available for 522 patients.

e Information available for 517 patients.

f Information available for 446 patients.

g Information available for 448 patients.

h Kruskal Wallis’s H Test

i Information available for 444 patients.
