# Supplementary material for: PKD1 is a potential biomarker and therapeutic target in triple-negative breast cancer
Source: Oncotarget. 2018 May 1;9(33):23208–19. doi: 10.18632/oncotarget.25292 (PMC5955414; doi:10.18632/oncotarget.25292)
Supplement: Supplementary file 3 [file oncotarget-09-23208-s003.docx]

**Supplemental table 2: Relationship between *PRKD1* mRNA levels and classical clinical/biological parameters in the TNBC subgroup**

|  | Number of patients (%) | | | |
| --- | --- | --- | --- | --- |
|  | Total population (%) | Low *PRKD1* mRNA levels | High *PRKD1* mRNA levels | Total population (%) |
| *Total* | 102 (100) | 51 (50.0) | 51 (50.0) |  |
| *Age*  ≤50  >50 | 39 (38.2)  63 (61.8) | 22 (56.4)  29 (46.0) | 17 (43.6)  34 (54.0) | 0.31 (NS) |
| *SBR histological grade* b,c  I  II  III | 3 (3.1)  18 (18.8)  75 (78.1) | 1 (33.3)  5 (27.8)  41 (54.7) | 2 (66.7)  13 (72.2)  34 (45.3) | 0.10 (NS) |
| *Lymph node status* d  0  1-3  >3 | 48 (47.1)  39 (38.2)  14 (13.7) | 28 (58.3)  15 (38.5)  7 (50.0) | 20 (41.7)  24 (61.5)  7 (50.0) | 0.46 (NS) |
| *Pathological size* d  ≤25mm  >25mm | 40 (39.7)  61 (60.3) | 18 (45.0)  32 (52.5) | 22 (55.0)  29 (47.5) | 0.18 (NS) |
| *KI67 mRNA expression* e,f  Median | 23.8 (8.6-117.2) | 21.2 (5.7-117.2) | 20.4 (0.8-106.7) | 0.49 (NS) |
| *Histological subtypes* g |  |  |  |  |
| Apocrine | 2 (3.0) | 0 | 2 (100.0) | 0.26 (NS) |
| Ductal | 59 (89.4) | 29 (49.2) | 30 (50.8) |  |
| Medullary | 4 (6.1) | 3 (75.0) | 1 (25.0) |  |
| Metaplastic | 1 (1.5) | 1 (100.0) | 0 |  |

a χ2Test

b Scarff Bloom Richardson classification.

c Information available for 96 patients.

d Information available for 101 patients.

e Information available for 68 patients.

f Kruskal Wallis’s H Test

g Information available for 66 patients.
