# Supplementary material for: PKD1 is a potential biomarker and therapeutic target in triple-negative breast cancer
Source: Oncotarget. 2018 May 1;9(33):23208–19. doi: 10.18632/oncotarget.25292 (PMC5955414; doi:10.18632/oncotarget.25292)
Supplement: Supplementary file 4 [file oncotarget-09-23208-s004.docx]

**Supplemental table 3: Inhibition of estrogen-independent clonogenicity of MCF7-PKD1 cells by the 41 PKD1 inhibitors**

| Molecule | IC50 (µM) |
| --- | --- |
| **AB9539** | **0.23** |
| AB8870 | 0.62 |
| AB8857-3 | 0.74 |
| AB8874 | 0.79 |
| AB9324 | 0.86 |
| AB9358 | 0.87 |
| AB9331 | 0.95 |
| AB9431 | 0.96 |
| AB9576 | 1.12 |
| AB8845 | 1.16 |
| AB8862 | 1.17 |
| **AB9275** | **1.27** |
| AB9428 | 1.31 |
| AB9429 | 1.39 |
| AB8424 | 1.41 |
| AB8823 | 1.47 |
| AB9225 | 1.95 |
| AB9452 | 1.96 |
| AB8905 | 2.29 |
| AB8912 | 2.44 |
| AB9282 | 3.01 |
| AB9185 | 3.16 |
| AB8908 | 3.25 |
| AB9323 | 3.40 |
| AB9129 | 3.71 |
| AB8831 | 4.11 |
| AB9145 | 4.91 |
| AB9270 | 4.94 |
| AB9297 | 5.02 |
| AB8909 | 5.15 |
| AB9195 | 5.47 |
| AB9130 | 5.55 |
| AB8899 | >10 |
| AB8910 | >10 |
| AB9109 | >10 |
| AB9110 | >10 |
| AB9118 | >10 |
| AB9137 | >10 |
| AB9159 | >10 |
| AB9367 | >10 |
| AB9376 | >10 |
